# A P300 Brain-Computer Interface Paradigm Based on Electric and Vibration Simple Command Tactile Stimulation

**DOI:** 10.3389/fnhum.2021.641357

**Published:** 2021-04-14

**Authors:** Chenxi Chu, Jingjing Luo, Xiwei Tian, Xiangke Han, Shijie Guo

**Affiliations:** ^1^Institute of Artificial Intelligence (AI) and Robotics, Academy for Engineering and Technology, Fudan University, as well as Engineering Research Center of AI & Robotics, Ministry of Education, Shanghai, China; ^2^Guanghua Lingang Engineering Application and Technology R&D (Shanghai) Co., Ltd., Shanghai, China; ^3^Jihua Laboratory, Guangzhou, China; ^4^Department of the State Key Laboratory of Reliability and Intelligence of Electrical Equipment and The Hebei Key Laboratory of Robot Perception and Human-Robot Interaction, Hebei University of Technology, Tianjin, China

**Keywords:** brain-computer interface, vibro-tactile stimuli, electro-tactile stimuli, P300 paradigm, spatial-frequency paradigm

## Abstract

This paper proposed a novel tactile-stimuli P300 paradigm for Brain-Computer Interface (BCI), which potentially targeted at people with less learning ability or difficulty in maintaining attention. The new paradigm using only two types of stimuli was designed, and different targets were distinguished by frequency and spatial information. The classification algorithm was developed by introducing filters for frequency bands selection and conducting optimization with common spatial pattern (CSP) on the tactile evoked EEG signals. It features a combination of spatial and frequency information, with the spatial information distinguishing the sites of stimuli and frequency information identifying target stimuli and disturbances. We investigated both electrical stimuli and vibration stimuli, in which only one target site was stimulated in each block. The results demonstrated an average accuracy of 94.88% for electrical stimuli and 95.21% for vibration stimuli, respectively.

## Introduction

Brain-Computer Interfaces (BCIs) provide a direct communication pathway between human brains and the external environment by recognizing voluntary changes in users’ brain activity, independent of the activity of peripheral afferent nerves or muscles (Dey et al., 2015). This technology can benefit disabled patients with difficulties in forming expressions by speaking or with body movements, such as patients with amyotrophic lateral sclerosis (ALS), muscular dystrophy, or locked-in syndrome (LIS), to communicate with the external environment (Birbaumer and Cohen, 2007; Chatelle et al., 2012; Naci et al., 2012). Moreover, BCI is an essential approach for patients to achieve motor and cognition rehabilitation for patients with diseases disrupting the neural pathways between the brain and the external environment, such as stroke, cerebral palsy, and atresia syndrome, which seriously affect the patients’ survival ability and quality of life (Wolpaw et al., 2002; Jolley et al., 2018).

Studies on BCI systems have a variety of paradigms for control, including Motor Imagery (MI) BCIs (Scherer et al., 2004; Nijholt et al., 2008; Demirer et al., 2009; Carlson and Millan, 2013; Meng et al., 2016) as spontaneous systems, visual BCIs (Farwell and Donchin, 1988; Cao et al., 2012; Falzon et al., 2012; Chen et al., 2015), auditory BCIs (Hill et al., 2005; Furdea et al., 2009; Klobassa et al., 2009; Guo et al., 2010; Halder et al., 2010; Kim et al., 2011; Xu et al., 2013), and tactile BCIs as evoked systems. Among the above systems, MI BCIs and visual BCIs are relatively widely studied and applicated, however, they are not a panacea. For example, many people are “MI blind” (Yao et al., 2017) or cannot efficiently generate imaginary motion instruments in the central nervous system (CNS). Besides, MI can easily cause brain fatigue, dizziness, nausea, and other adverse reactions. Visual BCIs have similar problems. Most visual BCIs are based on flickering stimuli, and the continuous flickering can lead to visual fatigue thus reduces users’ comfort (Punsawad and Wongsawat, 2012). It also has limitations to specific patients, such as those with atresia syndrome who lost their visual functions partially or entirely (Murguialday et al., 2011). Auditory BCIs are not widely used because of their susceptibility to environmental interferences and relatively low accuracy. Recently, much attention has been paid to tactile somatosensory stimulation for the following advantages: (1) it is easy to trigger and to generate ideal target signals without repeated training; (2) it does not impose an additional visual or audiovisual burden on the user; and (3) it is concealed, thereby not attracting the attention of others and helping to protect the personal privacy of the user.

A lot of online tactile BCIs have been designed and it has been demonstrated that an online system can achieve the same classification accuracy as an offline system (Rutkowski et al., 2015; Chen et al., 2020; Jin et al., 2020; Ziebell et al., 2020). Like visual and auditory BCIs, tactile BCIs can also perform multi-classification tasks. However, Ortner et al. (2014) demonstrated that a multi-classification task has lower classification accuracy compared to a two-classification task although it enables more information transmission. For a rehabilitation robot for people with relatively slow mental action, such as the stroke, atresia patients as well as the elderly, obtaining accurate motion intention is more important than information transmission. However, tactile BCI systems still have shortcomings, such as too many stimulators and relatively cumbersome operations (Chen et al., 2020; Jin et al., 2020; Ziebell et al., 2020). Furthermore, classification accuracy and speed for understanding the motion intention of the user still need to be improved for practical use.

In this paper, a new P300 tactile-BCI paradigm is proposed, and a classification algorithm is developed by combining spatial and frequency characteristics. P300-based BCI has the clear advantage of requiring no initial training. Tests were performed on 20 subjects by stimulating the pads of the index fingers of the left and right hands, with only one target pad being stimulated in each block. Both electrical and vibration stimulation were studied and compared.

## Materials and Methods

### Subjects

The study recruited 20 healthy subjects for tests, including 10 males and 10 females. Their ages were within the range of 20–30 years old, all right-handed. None of them had previously participated in a BCI experiment. They did not have neurological or psychiatric abnormalities or major medical conditions such as heart disease, had not recently taken any medication for neurological conditions, and were not dependent on alcohol or addictive drugs. Each subject was informed in detail of the purpose and precautions before the experiment with a signed informed consent form.

### Setup

**FIGURE 1:**
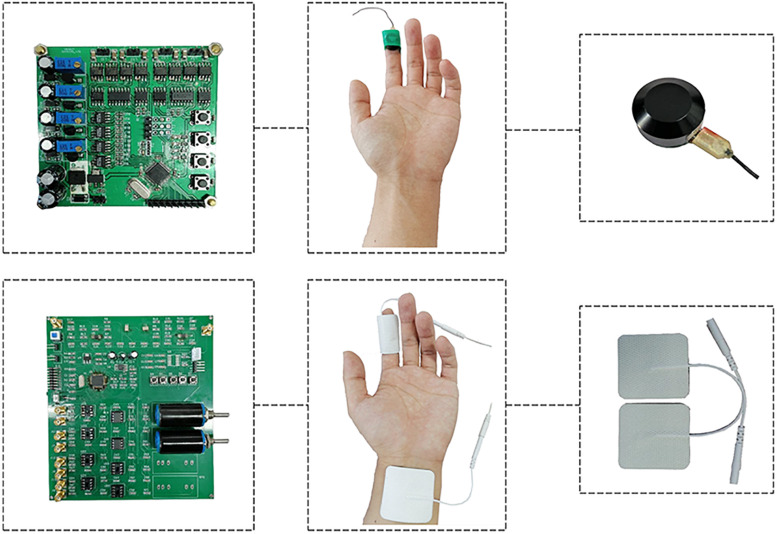
Devices for generating electrical and vibration stimuli: (1) Vibration stimulus trigger device on the top row: Four-channel DC motor drive (module STM32F103) with a rated power of 2 W. The driving voltage was adjustable from 0 to 5 V, and the vibration frequency was able to be changed from 0 to 300 Hz. (2) Electrical stimulus trigger device on the bottom row: Dual-channel STM32F103 arbitrary waveform generation module with a 12 V DC power supply. The output voltage could be adjusted from 2 to 12 V, and the output frequency was within the range of 0–1,000 Hz.

Devices for generating electrical and vibration stimuli were developed, as shown in Figure 1, and the parameters were set as follows:

(1) Vibration stimulus trigger device: Four-channel DC motor drive (module STM32F103) with a rated power of 2 W. The device was powered by an independent power supply. The driving voltage was adjustable from 0 to 5 V, and the vibration frequency was able to be changed from 0 to 300 Hz. The stimulus takes the form of square waves.
(2) Electrical stimulus trigger device: Dual-channel STM32F103 arbitrary waveform generation module with a 12 V DC power supply. The device was powered by an independent power supply. The output voltage could be adjusted from 2 to 12 V, and the output frequency was within the range of 0–1,000 Hz. The stimulus takes the form of sine waves. Besides, the maximum current of the device was much less than 5 mA which was below the human safety limit of current (Nave and Nave, 1985). In addition, safety measures were taken by introducing a limit circuit to ensure that the voltage and current do not exceed the safety limit.

### Paradigm Design

In this study, a new paradigm using only two types of stimuli was designed, differed from traditional dual classification tasks. The latter has the same proportion of the target stimuli and the disturbances, making it difficult to highlight the “low probability” of the target. The task we designed can be easily adopted, as only one targeted stimulation was implemented with “low probability” in each block. This paradigm is proposed to achieve high distinguishing accuracy, targeting the elderly and patients who are less able to learn and focus for long periods.

The paradigm proposed here was an improved Oddball paradigm. There were three stimuli modes: the left-hand target stimuli, the right-hand target stimuli, and disturbances. The feasibility and validity of the paradigm were verified by applying both electrical and vibration stimulation.

Since the Pacinian and Meissner mechanoreceptors of human bodies are sensitive to vibrations of frequencies in the range of 20–50 Hz and higher than 100 Hz (Breitwieser et al., 2012). The target stimuli frequency was set as 100 Hz with a duration of 150 ms, the disturbances frequency was set to be 23 Hz with a duration of 200 ms, and intervals between were set to 400 ms. No significant difference in BCI performance was noted between tactile stimulation on finger pads or the wrist, but studies showed that the former has a broader and more stable event-related desynchronization (Missiroli et al., 2019). Therefore, the index finger pads of the two hands were chosen as stimulation sites.

### Protocol

**FIGURE 2:**
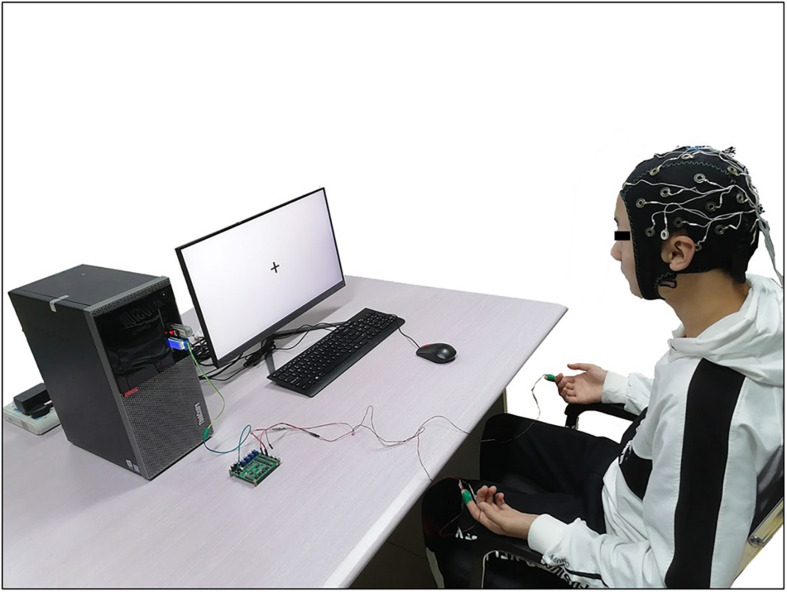
The test process: Each subject was asked to sit in a chair during the test in a relaxed condition, with their hands being rested on armrests. During the test, the subject should gaze fixedly at the “+” (symbol) on the screen.

Each subject was asked to sit in a chair during the test in a relaxed condition, with their hands being rested on armrests. The stimulators were attached to the pads of the index fingers, one on the left and one on the right. During the test, the subject gazed fixedly at the “+” (symbol) on the screen, with no eye movement, as shown in Figure 2. Vibration and electrical experiments were performed separately with a break time of 3 min.

**FIGURE 3:**
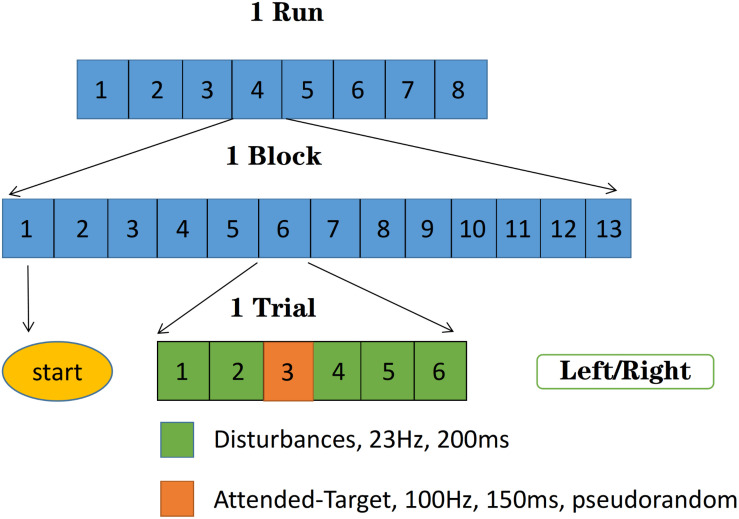
Experimental procedure: The target stimuli were 100 Hz with the duration of 150 ms, the disturbances were 23 Hz with the duration of 200 ms. Each run consisted of eight blocks with only one target site (left or right) stimulated in one block, and each block contained 13 trials. Each trial contained six stimuli, in which the only one target stimulus was selected pseudorandomly, and others were disturbances. The first trial in each block was all six disturbance stimuli to mark the start of the block.

Figure 3 shows the experimental procedure. The target stimulus was 100 Hz with the duration of 150 ms, the disturbances were 23 Hz with the duration of 200 ms. Each trial contained six stimuli, in which only one target stimulus was selected pseudorandomly, and others were disturbances. Each block contained 13 trials, and each run consisted of eight blocks with only one target site (left or right) stimulated in one block. The first trial in each block was all six disturbance stimuli to mark the start of the block. The subject was asked to focus on either the left or the right hand in one block, covertly counting the appearance of the target stimuli to enhance attention. There was a 1 min break after each block to avoid fatigue.

### Collection of EEG Signals Subjects

**FIGURE 4:**
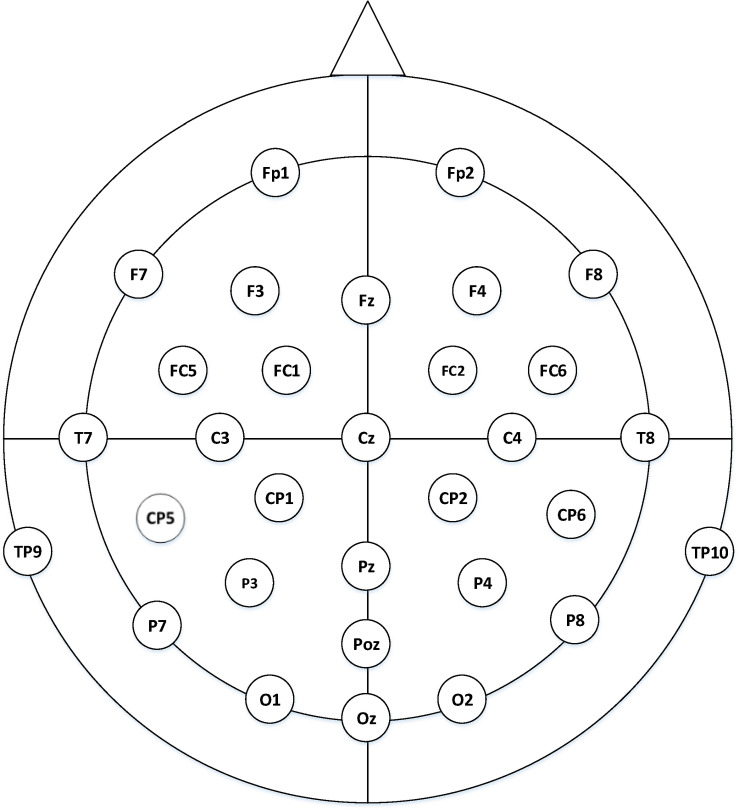
Sensor position in the EEG experiments: thirty-two electrodes were used based on the extended international 10–20 system, of which the ECG electrode was excluded.

EEG signals were collected using a 32-conductor electrode cap (BrainAmp^TM^ MR, Germany) following the 10–20 international standard, which was shown in Figure 4. The sampling frequency was set as 1,000 Hz. The reference electrodes for each channel were placed on the left and right mastoids, and the grounding electrodes were located between the Fz and Fpz electrodes. The impedance of an electrode was confirmed to be lower than 5 kΩ. Experiments were conducted in an electromagnetically shielded room to avoid noise and electromagnetic interferences.

### Data Description and Preprocessing

We collected data from 20 healthy subjects, three sets of which were abandoned because electrodes were not fixed well to the heads. Among the 17 subjects, there were 8 males and 9 females. MATLAB-EEGLAB toolkit (Delorme and Makeig, 2004) was used to preprocess the data, using the MNI (Montreal Neurological Institute) standard to determine the position of the electrodes. The ECG (Electrocardiogram) signals were removed, taking the average of TP9 and TP10 as a reference.

For the preprocessing, a 0.5–30 Hz bandpass filter was used to remove power frequency interference and most of the high-frequency noise. The initial 1,000 ms of the EEG following each target stimulus onset was extracted, with the baseline of 200 ms before onset. Independent component analysis (ICA) was conducted to remove EOG (Electrooculogram) signals, for example, components that distribute mostly in the prefrontal region, and components that distribute symmetrically, both with high energy in the low-frequency bands.

### Feature Extraction and Selection

We analyzed the energy distribution of the data through power spectral density (PSD), time-frequency analysis, event-related spectral perturbation (ERSP) and inter-trial coherence (ITC) analysis (Makeig, 1993), and applied a frequency restriction of 0.5–20 Hz. The frequency band was further segmented and bandpass filters were designed considering δ (0.5–3 Hz), θ (4–7 Hz), α (8–13 Hz), and β (above 14 Hz) waves, and the characteristics of the various frequency bands were extracted by a fourth-order Butterworth filter.

The volume conduction inbuilt from the multichannel electroencephalogram (EEG) recorded a blurred picture of brain activity (Blankertz et al., 2007). If the signal of interest is weak and other sources (including artifacts) produce strong signals in the same frequency range, this may seriously interfere with the EEG signal of interest. Especially during a real-time analysis of a single trial, this type of signal interference can be particularly serious (Baykara et al., 2016). Methods commonly used for improving the results of a single trial include (1) obtaining the required signal through repeated training (Baykara et al., 2016; Halder et al., 2016; Herweg et al., 2016) and (2) matching the system according to the individual characteristics of each user. In this study, Common Spatial Patterns (CSP) (Blankertz et al., 2007) adopted individual parameters that were used for spatial filter calibration. This allows us to improve single-trial classification significantly and to achieve high identification precision without superposing multiple trials.

The method used by the CSP algorithm is based on the simultaneous diagonalization of two covariance matrices. The signals before and after the spatial filter are expressed by E and Z, respectively, and are related by:

(1) Z=WE

where E is a matrix representing the raw EEG measurement data of a single trial, in which N represents the number of channels, T represents the number of measurement samples per channel, and W represents the CSP projection matrix. In this study, the spatial filter was constructed using the largest and smallest six features. Therefore, the first and last six rows of Z, i.e., Zp, p{1,2,…,6}, formed the feature vector Xp given in (2) as the input to a classifier.

(2) $$X_{p}=\log\left(var\left(Z_{p}\right)/\sum_{i=1}^{2m}var\left(Z_{p}\right)\right)$$

The operation has reduced the data dimension and thus the computation time. Traditional machine learning classifiers on a two- classification task were used in this study. Classifications were done using LDA (Linear Discriminant Analysis) and SVM (Support Vector Machine) (svc, c = 0.4, kernel: RBF) in a Python environment, and the results were validated by 10-fold cross-validation. For the sake of presenting our results better, we chose LDA for a further explanation.

On the other hand, information transfer rate (ITR) is a crucial indication of BCI performance, which is defined as the number of bits transmitted per unit time. The ITR is calculated as follows (Serby et al., 2005): ITR = BM where M is the mean number of decisions per minute, was set to 9.5238 (each trial was 3.15 s) and B is the number of bits per trial, which is given by:

(3) $$B=\log_{2}N+P\log_{2}P+\left(1-P\right){\text{log}}_{2}\left[\left(1-P\right)/\left(N-1\right)\right]$$

in which P represents the probability of accurate classification, was set to 0.95 and N the number of command categories, i.e., the number of classified categories was set to 2.

## Results

### ERP Components

**FIGURE 5:**
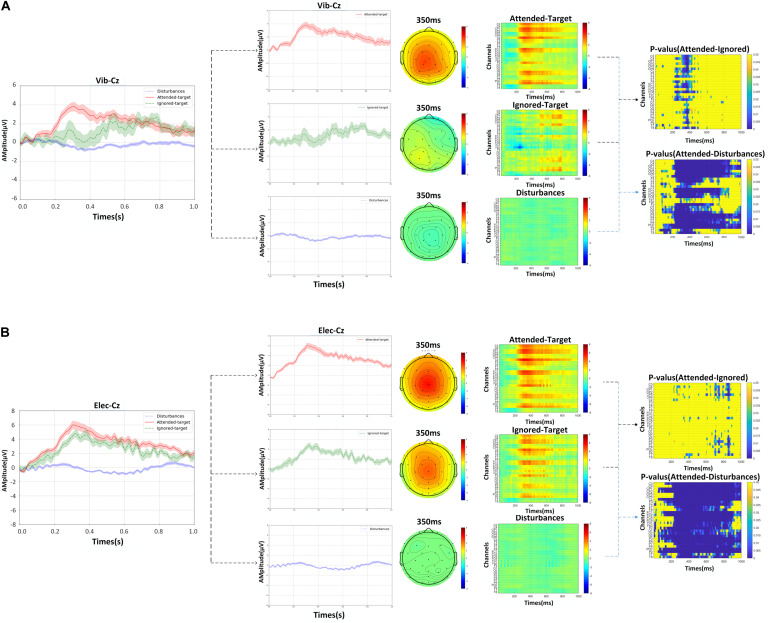
The grand averaged waveforms with error-shaded bar (with the standard error), corresponding scalp topographies at a latency of 350 ms, the grand averaged ERPs responses from EEG channels for attended-targets, ignored-targets and disturbance stimuli at Cz over all subjects, and *p*-values of the amplitude differences between the ERPs of response to attended-target and ignored target stimuli as determined by the ANOVA: **(A)** the data of vibration, **(B)** the data of electricity.

Under the attended-target stimuli, the characteristics of P300 were presented, while no peaks could be observed for the disturbance stimuli, i.e., ERP signals were not generated. Figure 5 shows the superimposed average of 12 trials in all blocks of all subjects and represents the EEG signals evoked by attended-targets and ignored-targets and disturbance stimuli. This demonstrated the effectiveness of P300 using the proposed paradigm. Corresponding scalp topographies at a latency of 350 ms were also shown in the figure, and sensors relating activated regions under target stimuli were significantly higher than in other areas.

### Selected Features and Contributions

**FIGURE 6:**
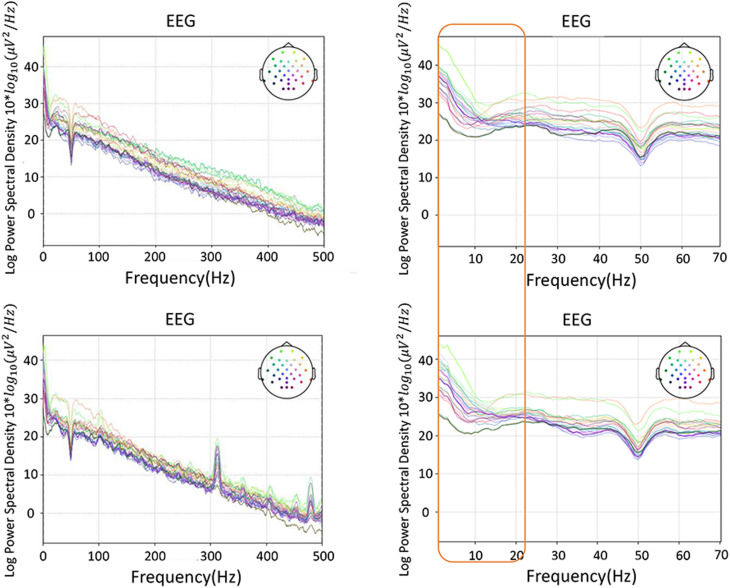
A representative of PSD-based spectrum analysis, with the most frequency information in 20 Hz: the top row is the vibration task, the bottom row the electrical task

**FIGURE 7:**
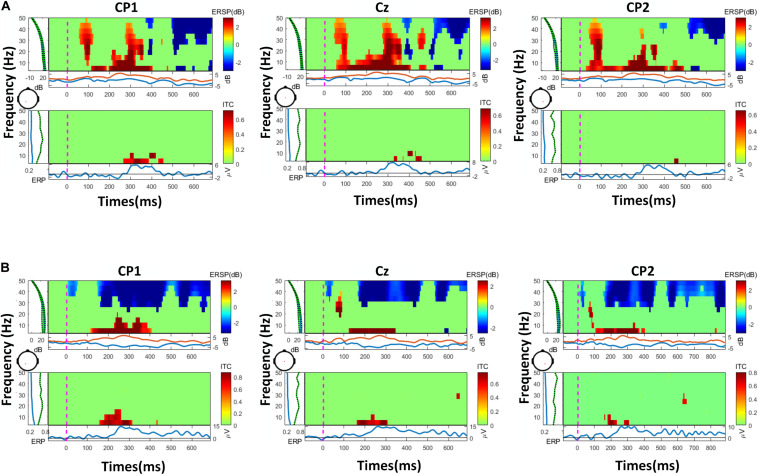
The results of ERSP (event-related spectral perturbation) and ITC (inter-trial coherence)of two representatives, with the most frequency information in 20 Hz: **(A)** a representative of the vibration task, **(B)** a representative of the electrical task.

The PSD-based analysis, event-related spectral perturbation (ERSP) and time-locked inter-trial coherence (ITC) analysis of a representative were performed on the data, as shown in Figures 6, 7. The frequency information was densely concentrated within 0–20 Hz. The 20 Hz range of internal frequencies was then divided according to δ, θ, α, and β waves. To investigate the impact of different frequency bands on the classification effect, a sliding time window with a length of 500 ms and a step length of 100 ms was used for real-time performance evaluation and visualization.

**FIGURE 8:**
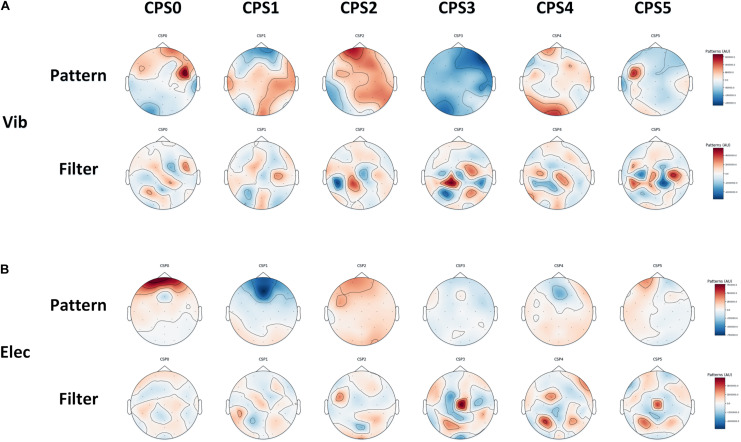
Six spatial patterns and corresponding spatial filters on average of all subjects we choose using CSP, which can be used to extract spatial features from the brain: **(A)** the vibration task, **(B)** the electrical task.

Further analysis found that θ, α, and β waves all had different weights on the performance of the classification model in populations, a contrasting example of two representative subjects is shown in Figure 8. These three wavebands reflect, respectively, three states of a human being: (1) the subconscious state of deep relaxation and no stress (4–7 Hz), (2) the optimal brain state for learning and thinking when the mind is awake, but the body is relaxed, providing a “bridge” between the conscious and subconscious (8–13 Hz), (3) the state of being stressed or mentally tired (14–20 Hz). The diversity among people and wavebands reflected the contribution of various psychological states to classification during the experiment. Moreover, in this model, δ waves (0.5–3 Hz) made hardly any contribution to classification performance (contributing less than the average chance level). Therefore, we chose a band of 4–20 Hz for classification.

### Features Extracted Through CSP

**FIGURE 9:**
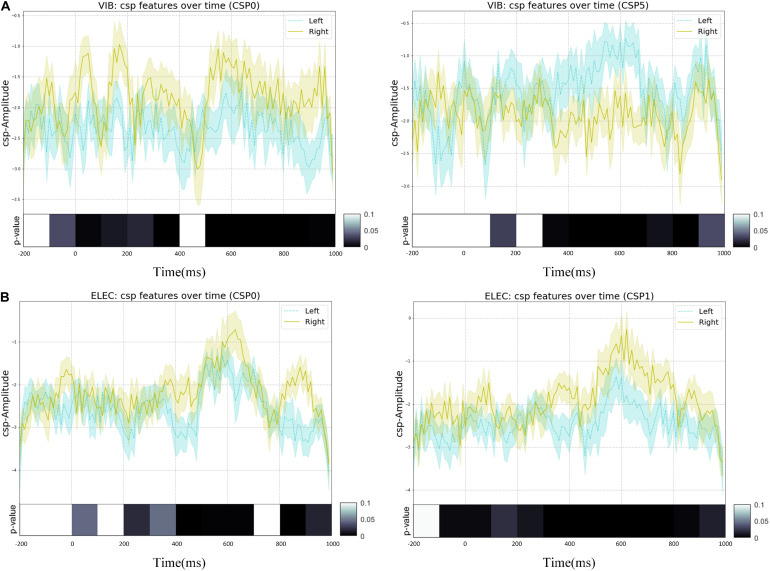
The most representative EEG features over all subjects were transformed by the selected spatial filters of CSP with error-shaded bar (with the standard error), of which one feature point was extracted from the real EEG signals in every 10 ms, and the *p*-values of left-handed and right-handed feature vectors were calculated in every 10 feature points (i.e., every 100 ms), which was verified by paired *t*-test: **(A)** the vibration task, **(B)** the electrical task.

**FIGURE 10:**
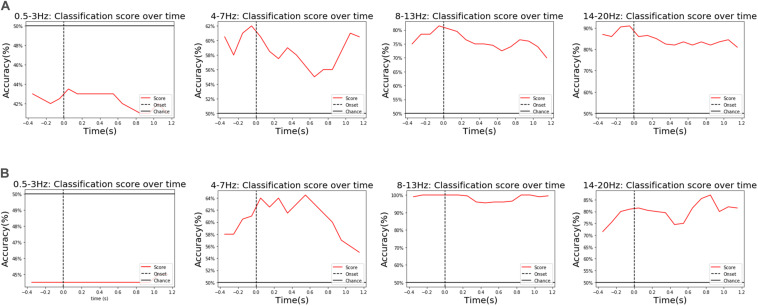
An example of the diverse contributions for different subjects and wavebands. For example, a subject in the vibration task in **(A)** showed that β waves (above 14 Hz) had the greatest influence on classification performance, followed by α waves (8–13 Hz), while another subject also in the vibration task in **(B)** showed that α waves (8–13 Hz) had the greatest influence on classification performance, followed by β waves (above14 Hz).

In this study, the spatial filter was constructed using six of the largest and smallest features. The corresponding spatial patterns and spatial filters we constructed by CSP on average of all subjects were shown in Figure 9. The most representative filtered features extracted through CSP of the right hand and the left hand on average of all subjects was shown in Figure 10, of which one feature point was extracted from the real EEG signals in every 10 ms, and the *p*-values of the feature vectors of both were calculated in every 10 feature points (i.e., every 100 ms), which was verified by paired *t*-test. It shows that through CSP spatial filters, features of the left hand and the right hand can be directly separated in the time domain, which may not be easily distinguished before.

### Classification Results

**FIGURE 11:**
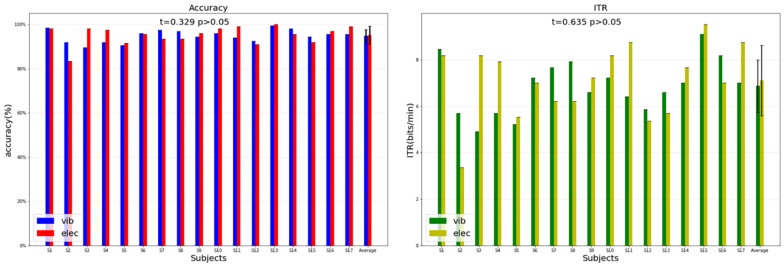
The classification accuracy and ITR of the two stimulation patterns over all subjects.

**FIGURE 12:**
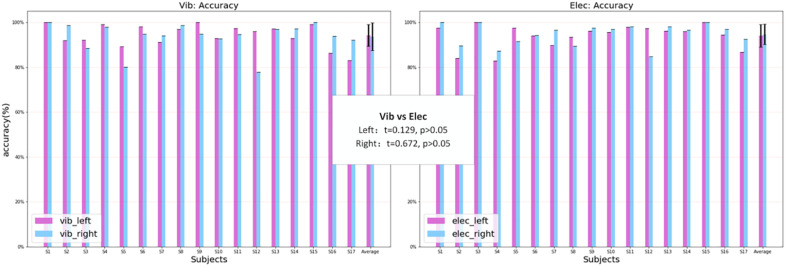
The single-target classification accuracy over all subjects for the vibration and electrical mode.

The classification result was from the data of the left attended-targets and the right attended-targets. Under vibration stimuli, the highest classification accuracy and ITR was 98.50% and 9.09 bits/min, the lowest was 89.50% and 4.91 bits/min, and the average was 94.88% ± 2.85% and 6.75 ± 1.17 bits/min. Under electrical stimuli, the highest classification accuracy was 100% and 9.52 bits/min, the lowest was 83.5% and 3.37 bits/min, and the average was 95.21% ± 4.10% and 6.88 ± 1.56 bits/min. Figure 11 shows the accuracy of the classification and the ITR under vibration and electrical stimuli of the P300 signals over all subjects. The statistical analysis showed that there was no significant difference in the classification accuracy between the two modes, and the ITR of both modes was sufficient to meet the information transmission requirements for the control of a rehabilitation robot for the elderly. In the vibration mode, 9 out of 17 subjects achieved higher accuracy when the target stimuli were delivered to the left hand than the right hand, which was counted 7 out of 17 subjects (Figure 12 left). While in the electrical mode, 12 out of 17 subjects achieved higher accuracy when the target stimuli were delivered to the right hand than the left hand, which was counted 3 out of 17 subjects (Figure 12 right). The statistical analysis of the single-target classification accuracy showed no significant difference between two target sites in both modes, and also no significant difference of the single-target classification accuracy between the two modes (*p* > 0.05).

**FIGURE 13:**
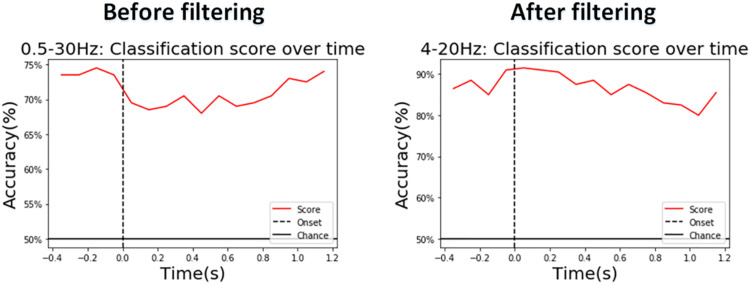
A representative in the vibration task: A sliding-time window with a length of 500 ms and a step length of 100 ms used for real-time performance evaluation before and after the frequency band we chose.

We also show the real-time classification performance of the proposed model before and after the selected waveband filter (both through the CSP spatial filter) with the LDA classifier on a representative (Figure 13). After our selected filter, the classification accuracy was significantly high at the beginning with an accuracy of around 90%, and the lowest accuracy was around 80% which occurred 1 s around after the start time.

### The Role of Attention

The amplitude of the ERPs can be significantly improved with the attention attended as it was shown at the latency of 350 ms. In Figure 5, we also contrasted waveforms, the corresponding responses, and *p*-values of ERPs among the attended, ignored, and disturbance stimuli. Besides, the grand averaged ERPs’ responses observed from EEG channels over all subjects were also showed more intensely with the attention on, especially in the channels C3, C4, CP1, CP2, Cz, F3, F4, FC1, FC2, Fz, P3, P4, POz, Pz. This further confirmed that the proposed paradigm with attention can produce a significant increase in the performance on P300, which was conducive to improving classification.

## Discussion

### ITR, ACC, and Categories

The main advantage of tactile-BCIs is to avoid burdening the visual or auditory system and are concealed. Nevertheless, its disadvantages are also obvious, for example, low accuracy and ITR have affected its applicability. In order to improve the ITR, more complex multi-classification tasks was designed, for example, Ortner et al. (2014) developed a two-classification task with stimulators on both wrists and back, as well as an eight-classification task with stimulators on both fingers, which was experimented in 12 healthy subjects and 6 patients with LIS and verified the feasibility of the vibration-BCIs in both healthy ones and patients with brain damage. It was also convinced that the multi-classification task had a higher ITR than the two-classification-task, though of which the accuracy was lower. However, the accuracy of intention recognition may be much more important than ITR for the elderly and patients with ALS, LIS, strokes, etc. who are relatively tardy and have lower demand for quick responses.

### The Existing Tactile BCIs

The reason that the performance on the accuracy of two-classification tasks was always less promising than other modes may be the indistinguishable “small probability” of the target-stimuli. Some instances were listed to better instruct the “small probability” we were concerned about. Brouwer and van Erp (2010) designed a lumbar body-sensing vibration paradigm conducted on 11 healthy subjects, which achieved an accuracy of 73% in the two-classification task. Ortner et al. (2014) achieved an accuracy of 80% in the two-classification task with three tactile-stimulators on both wrists and back, Guger et al. (2017) proposed a two-classification task using three tactile-stimulators on both wrists and shoulder, and achieved an accuracy of 86.7%, however, 12 trails were needed to generate an instruction costing 38 s, which is too long for control. The paradigms mentioned above may not well emphasize the “small probability” of the target-stimuli, which was paid much attention to in our paradigm on the contrary, thus leading to a more ideal result.

### Benefits of Somatosensory Stimulation

Somatosensory input of the tactile-stimuli has been found to increase motion-related cortical excitability in both healthy subjects (de Moraes Silva et al., 2015; Lapole and Tindel, 2015; Lopez et al., 2017) and stroke patients (Rothwell and Rosenkranz, 2005; Marconi et al., 2011). It has been clinically proven that the combination of somatosensory stimuli and motor commands had the potential to improve hand functions after stroke (Conforto et al., 2010; Fleming et al., 2015). Meanwhile, the accuracy of BCI control was positively correlated with the rehabilitation efficacy (Ramos-Murguialday et al., 2013). Therefore, it is of great significance for rehabilitation in the elderly and patients with ALS, LIS, strokes, etc. that we proposed the tactile-BCI with a simple and high accuracy paradigm.

### Discussions on Two Modes

In our paradigm, vibration and electrical modes were applied to investigate the validity of the proposed P300-paradigm. The average intention recognition accuracy for both modes was close to 95%, and some individuals even reached 100%. This meant that both vibration and electrical tactile stimulation under our paradigm could effectively evoke the P300 signal and well recognize the user’s intention. However, our experimental results suggested that there were also some aspects distinct from the two modes. For example, individual sensitivities to different tactile stimuli, some subjects had a better performance on one kind of stimuli rather than the other one, some had nearly the same sensitivity to both. Moreover, the stability among the subjects also reflected some differences, the performance of vibration stimuli seemed to be more stable than that of electrical ones (STD-vib = 0.0285, STD-elec = 0.0410), although electrical stimulation may show better performance for certain subjects.

Specifically, both modes have their own advantages and disadvantages. As for the vibration mode, vibration stimulation may lessen pain and reduce tremors, also soothes stiffness and increases dopamine levels in the brain (Mosabbir et al., 2020), which may help relieve symptoms. Vibration on muscle has also been shown to increase the corticospinal excitability assessed by transcranial magnetic stimulation (TMS) and to change voluntary force production in healthy subjects, and motor response generated with a descending cortical drive in chronic hemiparetic subjects can be increased during vibration (de Andrade Melo et al., 2015). Vibration is usually considered safe for most people, but people pregnant, having seizures, or having a pacemaker may not suit for this because it may cause resonance reactions. Moreover, noise pollution caused by vibration can also be a trouble.

As for the electrical mode, safe applications of electrical stimulation can be used to generate controlled limb movements in individuals who have sustained CNS injuries such as spinal cord injury (SCI) or stroke (Popovic et al., 2016). There are also changes showed in the spinal and cortical re-organization and benefits obtained after some electrical stimulation (Popovic et al., 2003, 2016; Thrasher et al., 2008; Kapadia et al., 2011), with therapeutic effectiveness accompanied by long-lasting re-organization in the brain and CNS (Shin et al., 2008; Sasaki et al., 2012; Carson and Buick, 2019; Milosevic et al., 2020). However, muscle fatigue is an obvious defect of the mode. It will affect the training duration which could delay patients’ recovery rate, and muscles will get fatigue at a different rate as the frequency increases (Naeem et al., 2013).

### Possibilities That Affecting Perfomances

Somatosensory capabilities can be affected with age (Wickremaratchi and Llewelyn, 2006), which maybe further reduce its applicability to the target population of tactile-BCIs. Tactual perception is mediated by the four mechanoreceptors: Pacinian corpuscles, Meissner’s corpuscles, Merkel’s discs and Ruffini endings all of which show increasing detection thresholds with age (Gescheider et al., 1994). Additionally, the sensitivity, accuracy and speed of the recognization for tactile patterns can also decrease (Cauna, 1964; Kok, 1999; Wickremaratchi and Llewelyn, 2006; Master et al., 2010) with an overall decrease in somatosensory capabilities (Gescheider et al., 1994). Moreover, the weaker attention and memory can also contribute to poorer performance on P300-BCIs (Kok, 1999).

Nevertheless, studies have shown that training can improve P300-BCIs’ performances (Baykara et al., 2016; Halder et al., 2016). Similarly, the defects that affect the performances of tactile-BCIs led by ages can also be improved through repeated training (Herweg et al., 2016; Eidel and Kübler, 2020), where the amplitude of the tactile-P300 signal was improved, as well as an amazing improvement in the accuracy and ITR after effective training. “Most notably we found no plateau for ERP amplitudes, area between curves, single-trial accuracy or ITR, suggesting participants may benefit from even more training.” (Herweg et al., 2016), which proved age-related tactile change can be remedied through training, thus the effects of age-related changes in tactile perception on BCI performance appeared to be both advantages and disadvantages.

## Conclusion

In this paper, a new P300 paradigm combining spatial and frequency information was proposed to solve the problems of existing tactile-BCIs, for instance, the low classification accuracy, overlapping average of multiple trials, and low efficiency. An adaptive algorithm was designed. The paradigm we proposed only needed two stimulators and had a simpler task to concentrate on the target ones for subjects, thus was probably more suitable for the elderly and patients with stroke, LIS, ALS, etc. Two tactile stimulation modes (vibration and electrical) were designed to verify the feasibility of the paradigm, and tests were conducted on 20 healthy subjects. Through the selection of specific frequency bands and an appropriate spatial filter with an enhancement of attention, both of the two tactile modes have achieved stable and excellent identification results for classification. The results demonstrated an average accuracy of 94.88% for electrical stimuli and 95.21% for vibration stimuli, respectively.

## Data Availability Statement

The original contributions presented in the study are included in the article/supplementary material, further inquiries can be directed to the corresponding author/s.

## Ethics Statement

The studies involving human participants were reviewed and approved by the Ethics Committee of the 983 Hospital of Joint Logistics Support Tianjin. The patients/participants provided their written informed consent to participate in this study.

## Author Contributions

CC and JL jointly completed the article writing and interpretation of algorithms. XT and XH participated in the early design and production of hardware stimulation devices. CC and XT participated in the paradigm design and recruitment of volunteers for the experiment. SG provided fund support of the project, academic guidance as the person in charge. All authors contributed to the article and approved the submitted version.

## Conflict of Interest

CC and SG were employed by company Guanghua Lingang Engineering Application and Technology R&D (Shanghai) Co., Ltd. The remaining authors declare that the research was conducted in the absence of any commercial or financial relationships that could be construed as a potential conflict of interest.
